# Raffinose and Hexose Sugar Content During Germination Are Related to Infrared Thermal Fingerprints of Primed Onion (*Allium cepa* L.) Seeds

**DOI:** 10.3389/fpls.2020.579037

**Published:** 2020-10-06

**Authors:** Meenakshi Thakur, Poornima Sharma, Anjali Anand, Vinod Kumar Pandita, Arti Bhatia, Suchitra Pushkar

**Affiliations:** ^1^Division of Plant Physiology, ICAR-Indian Agricultural Research Institute, New Delhi, India; ^2^Indian Agricultural Research Institute Regional Station, Karnal, India; ^3^Centre for Environment Science and Climate Resilient Agriculture, ICAR-Indian Agricultural Research Institute, New Delhi, India

**Keywords:** cytochrome c oxidase, emergence index, onion seeds, relative temperature profile, respiration, seed priming, soluble sugars profile

## Abstract

Priming is used to increase vigor, germination synchronization, seedling growth, and field establishment by advancing metabolic processes within seeds. Seed respiration is a good indicator of the metabolic processes that lead to transition toward germination. Onion seeds (cv. Pusa Ridhi) subjected to osmopriming (−1.5 MPa PEG6000 for 7 days), magnetopriming (100 mT for 30 min) and halopriming (150 mM KNO_3_ for 6 days), were evaluated at different times of imbibition to study the emergence index and respiration indices such as infrared thermal fingerprint, CO_2_ evolution rate, cytochrome c oxidase activity, and soluble sugars profile. Haloprimed seeds exhibited 42.5% higher emergence index as compared to unprimed control. Primed and unprimed seeds showed negative values for relative temperature (ΔT) (difference in temperature of seed and its immediate environment). Haloprimed seeds had the lowest values (−4.1 to −2.3°C) compared to other priming treatments over the germination period. Soluble sugars like raffinose, sucrose, glucose, and fructose contents were monitored and it was observed that *en masse* raffinose, glucose, and fructose levels were (17.5–59.9%) lower in haloprimed seeds over control. A positive correlation (*r*^2^ = 0.504^∗∗^) was derived between the amount of these sugars and ΔT. Seed respiration, measured as CO_2_ evolution rate was more for haloprimed seeds that indicated that these soluble sugars were used as respiratory substrates. Significantly higher cytochrome c oxidase activity (40.7–89.8% and 12.5–66.6%) was observed in all primed seeds at 28 and 36 h, respectively. Among the various seed priming methods, halopriming proved to be the most effective priming treatment in onion seeds as evidenced by the higher respiration indices that resulted in faster metabolic rate and emergence index.

## Introduction

Onion (*Allium cepa* L.) is an important vegetable crop grown and consumed widely across the world. Latest statistics report production of 22,071 × 10^3^ tonnes from an area of 1315 × 10^3^ ha in India, making India as the second-largest producer of onion in the world after China (FAO, 2018). Onion seeds encounter rapid loss in germination capacity and vigor during storage that manifests huge losses to seed companies all over the world (George, 1985) and increases the cost of seed for planting (Black et al., 2006). The reversible loss in vigor can be restored by pre-germination seed treatments (Nagarajan et al., 2012), therefore, prospecting for various methods of seed enhancement would help in salvaging the low vigor seeds.

Seed priming is an established method of seed enhancement which is potentially able to promote seed vigor and help in establishing a rapid and uniform crop stand. Seed priming can be done by various approaches such as hydropriming, osmopriming, chemical priming, hormonal priming, biological priming, solid matrix priming, magnetopriming, etc. (Thakur et al., 2019). Improvement in seed vigor is promoted by the activation of respiration and ATP production during the imbibition stage (Weitbrecht et al., 2011). It enhances the events in the early phase of germination by increasing the absorption of water, breakdown of reserve material that provide energy and carbon skeletons through increased respiratory metabolism, mitochondrial repair and multiplication (Bewley et al., 2013). Soluble sugars like raffinose, sucrose, glucose, and fructose are important carbon sources for the seeds during the germinative process as they are fast-use substrates for respiration and production of ATP for the growing seedlings (Koops and Groeneveld, 1990). Measurement of seed respiration and its related processes provides fast and accurate measurement of the metabolic advancement amongst the seed lots as oxygen consumption is directly proportional to the energetic potential of the seed.

Therefore, the present study was conducted to find a relationship between the pattern of sugar profile and infrared seed temperatures during imbibition of the differentially primed onion seeds, i.e., osmo-, magneto-, and halo-primed to understand their role in germination.

## Materials and Methods

### Seed Priming

Seeds of onion cv. Pusa Ridhi was procured from ICAR- Indian Agricultural Research Institute Regional Station, Karnal, India. The seeds were subjected to various priming treatments such as osmopriming (Dorna et al., 2013), magnetopriming (magnetic field treatment of 100 mT for 30 min) and halopriming after prior standardization in our laboratory. Seeds of uniform size were soaked in −1.5 MPa PEG 6000 for 7 days (osmopriming) and 150 mM KNO_3_ for 6 days (halopriming) in bottles with screw caps. After the respective soaking time for each treatment, the soaked seeds were thoroughly washed with distilled water, followed by drying at room temperature. The weight of seeds was taken at fixed intervals till their pre-priming moisture content (9%) was obtained. Magnetoprimed seeds were used as such for further experiments and unprimed seeds served as control. Emergence index (EI) was determined in the differently primed onion seeds after sowing in the field. Unprimed and differently primed onion seeds were imbibed between layers of germination paper and kept at a temperature of 20 ± 1°C in an incubator for different time intervals, i.e., 8, 28, 36, and 52 h. Samples were collected in triplicates for the determination of cytochrome c oxidase activity and soluble sugars profile in the imbibed seeds. Relative temperature measurements and carbon dioxide evolution rate studies were also conducted at different intervals of imbibition time.

### Emergence Index

A total of 50 onion seeds from each priming treatment along with control were planted in the field in 2 m rows and replicated thrice in a randomized block design (RBD). The data were recorded at regular intervals from the first count till the last count. Emergence index was calculated and expressed according to the formula given by Kader (2005) as:

Emergence⁢index⁢(EI)=(10×n1)+(9×n2)+⋯+(1×n10)

where, n1, n2,…, n10 = number of seeds germinated on the first, second and subsequent days until the 10th day; 10, 9,…, and 1 are weights given to the number of germinated seeds on the first, second and subsequent days, respectively.

### Infrared Thermal Fingerprint

The infrared thermal fingerprint of onion seeds at different imbibition intervals (8, 12, 16, 20, 24, 28, 32, 36, 52 h) was observed by using Infrared Thermographic camera (Model TESTO 890-2). Thermal images of seeds were acquired in the closed chamber keeping the surrounding conditions relatively constant over the time of imbibition. The camera was placed at a distance of 90 cm from the sample for about 2 h before the infrared measurements to allow the optics of the camera to reach thermal equilibrium with the air temperature. The results were recorded as relative temperature (ΔT) representing the difference in temperature of the seed and its immediate environment.

Ambient temperatures of both air and water were kept constant with minimal convection to minimize the temperature gradients between seeds. Sufficient space was also maintained between the adjacent seeds to prevent the interference due to the heat flow of individual seed. Temperature data for each onion seed was recorded by taking thermal images at different times of imbibition in three replications. To correct for temperature gradients between the seed and the surroundings, ΔT was calculated as the difference between the temperature of the seed and the zone affected by the thermogenic activity of the seed, ultimately providing the individual “seed calibration reference temperature.”

### Carbon Dioxide Evolution Rate

The rate of CO_2_ evolution was determined by gas chromatography (Shimadzu GC-8A). Fifty seeds from each priming treatment along with unprimed control were placed in triplicates over wet germination sheets in closed jars sealed with parafilm. Seeds were counted instead of weighing due to the reason that the number of individual embryos is important for the determination of respiration (Barton, 1945). The mean dry weight of the seeds at the start of the experiment amongst different treatments was 0.074 ± 0.003 g. The jars containing seeds were kept mechanically undisturbed in an incubator maintained at constant temperature (20°C) as imbibed seeds are very sensitive to temperature fluctuations. After several preliminary tests, a 52 h respiration period was shown to be satisfactory. The CO_2_ released was collected in syringes at different time intervals, i.e., 8, 28, 32, 36, 44, and 52 h. Empty closed jar along with wet germination paper served as a blank or negative control for measuring the CO_2_ present in the jar before the start of the experiment. The samples were analyzed for CO_2_ evolution rate using gas chromatograph fitted with a methanizer and a flame ionization detector. Nitrogen was used as a carrier gas and NIST traceable standards of CO_2_ of 300 and 600 ppm concentrations were used as standards. The CO_2_ evolution rate was expressed in μmol h^–1^ g^–1^ DW.

### Cytochrome c Oxidase Activity

Mitochondria were isolated from imbibed onion seeds according to the method given by Bonner (1967). Imbibed onion seeds (1 g) were homogenized at 4°C in ice-cooled grinding medium (1:10 w/v), containing a mixture of mannitol (0.3 M), EDTA (1 mM), BSA (0.1%), and cysteine (0.05%). Homogenate was centrifuged at 1000 × *g* for 15 min to collect the supernatant. The supernatant was again centrifuged at 10,000 × *g* for 15 min. The pellet, thus obtained was suspended in wash medium containing mannitol (0.3 M), EDTA (1 mM), and BSA (0.1%) followed by centrifugation at 250 × *g* for 10 min. The supernatant was collected and centrifuged at 6000 × *g* for 15 min. Pellets containing mitochondria were collected and suspended in wash medium. Cytochrome c oxidase activity in isolated mitochondrial fraction was determined according to the method of Wharton and Tzagoloff (1967). Cytochrome c oxidase activity was measured spectrophotometrically as the oxidation of cyt^*red*^ as indicated by the decrease of absorbance at 550 nm for 2 min. The total enzyme activity was expressed as M cytochrome c oxidized min^–1^ g^–1^ DW.

### HPLC Profile of Soluble Sugars

Sugars in imbibed onion seeds were quantified using HPLC by following the modified method of Karkacier et al. (2003). Imbibed onion seeds (0.2 g) were homogenized using HPLC water (1 ml) followed by shaking on a horizontal shaker for 15 min. The homogenate was centrifuged at 4000 × *g* for 5 min and the supernatant was collected. Acetonitrile (0.7 ml) was added to 0.5 ml supernatant followed by incubation at room temperature for 2 h to precipitate soluble proteins. The mixture was centrifuged at 3670 × *g* for 5 min and the supernatant was collected. The supernatant (0.1 ml) was evaporated to dryness in a hot air oven. The dried residue was dissolved in 0.1 ml 0.005 M sulfuric acid followed by filtration through 0.2 μm syringe filters and analyzed in Hiplex-H column (300 mm × 7.7 mm Agilent Technologies 1100/1200 Series, United States) maintained at 35°C with the refractive index detector with a flow rate of 0.6 ml min^–1^. The standards were included with each group of samples loaded to the HPLC at the same time as a control on detector response. The sugars contents were expressed as mg g^–1^ DW.

### Statistical Analysis

The data were analyzed by one-way analysis of variance (ANOVA) and *post hoc* multiple comparisons using SPSS version 16.0 (SPSS Inc., Chicago, IL, United States). Least significant differences were considered at *p* < 0.05.

## Results

### Emergence Index

**FIGURE 1:**
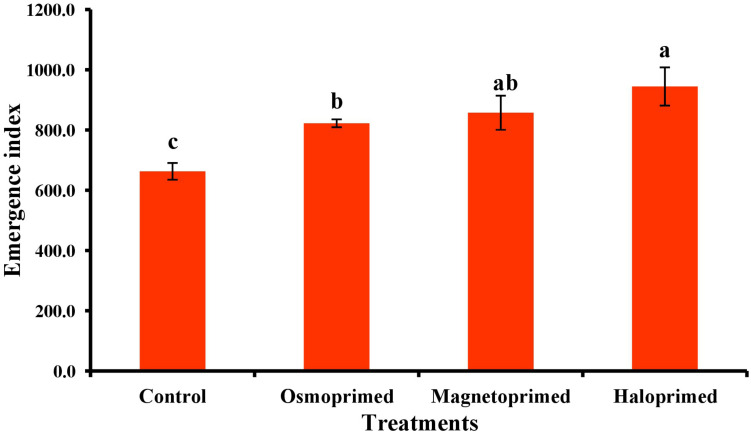
Emergence index of differently primed onion seeds. Data represent mean ± SE of four biological replications. Different letters over the bars indicate significant differences among means based on LSD test (*p* < 0.05).

Emergence index encompasses the germination percentage and speed of germination. All the treatments exhibited significantly higher EI than control. Haloprimed seeds exhibited a significant increase of 42.5% in EI value followed by magneto- (29.3%) and osmo-primed seeds (24.1%) as compared to unprimed seeds (Figure 1). Primed seeds with higher EI value suggest a higher percentage of seeds with an increased rate of germination.

### Fingerprint of Infrared Relative Temperature (ΔT) in Germinating Primed and Unprimed Seeds

**FIGURE 2:**
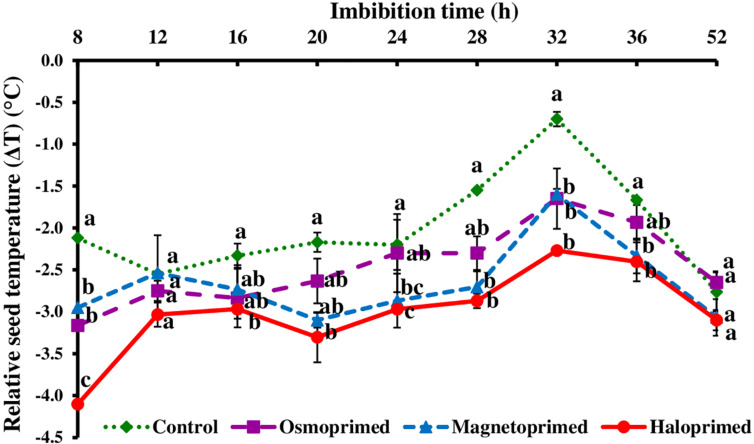
Relative seed temperature (ΔT) of differently primed onion seeds at different imbibition time. Data represent mean ± SE of three biological replications. Different letters indicate significant differences among means based on LSD test (*p* < 0.05). ΔT was calculated as the difference between seed temperature and the zone affected by the thermogenic activity of the seed, providing the individual “seed calibration reference temperature.”

**FIGURE 3:**
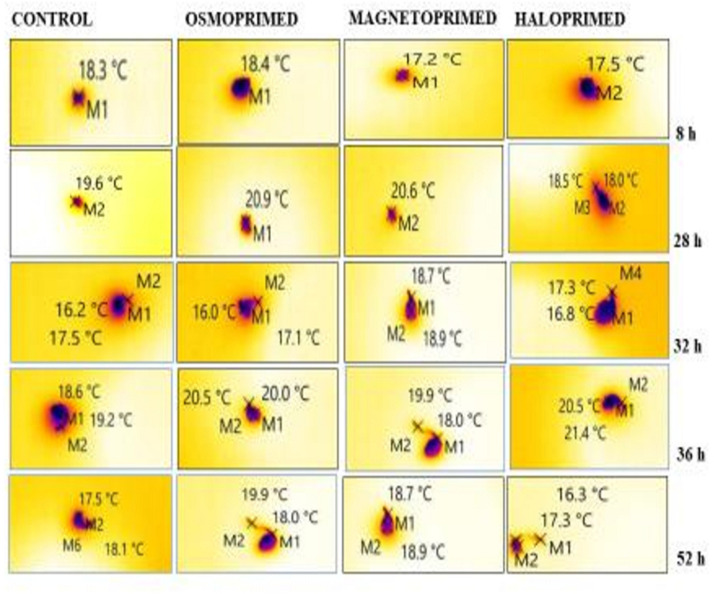
Representative thermal images of differently primed onion seeds at different imbibition time. “M” marks on the images represent the point selected for determining the temperature of the imbibed seed.

In osmo-, magneto-, and halo-primed seeds, relative temperature (ΔT) ranged from −3.2 to −1.7°C, −3.1 to −1.6°C, and −4.1 to −2.3°C, respectively. Relative temperature (ΔT) in control ranged from −2.8 to −0.7°C (Figures 2, 3). At 8 h, haloprimed seeds showed significantly low temperature as compared to other treatments. Magnetoprimed germinating seeds exhibited ΔT values similar to haloprimed seeds at 24 and 28 h, that was significantly more negative (30.4–35.0% and 74.2–85.2% lesser) than control, whereas with the progress of germination (32 h) the values of ΔT in osmo-, magneto-, and halo-primed treatments were significantly more negative than control. After 32 h a slight decline was observed in ΔT values in all the treatments up to 52 h. At 52 h, ΔT values in all the treatments were significantly *at par*.

### CO_2_ Evolution Rate

**FIGURE 4:**
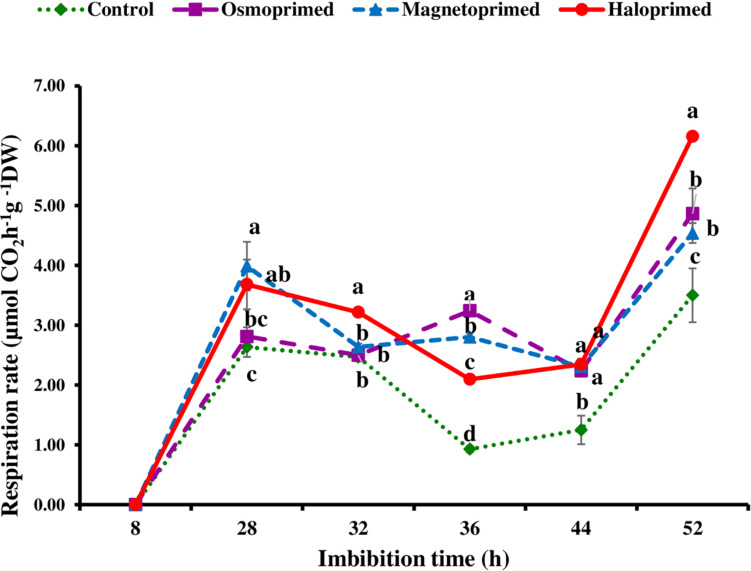
Respiration rate of differently primed onion seeds at different imbibition time. Data represent mean ± SE of three biological replications. Different letters indicate significant differences among means based on LSD test (*p* < 0.05).

Based on fingerprints of ΔT values, the respiration rate in primed and unprimed seeds was measured at the imbibition time showing higher fluctuations, i.e., at 8, 28, 36, and 52 h. All the treatments showed significantly higher respiration rate (6.7–51.4%) at 28 h of imbibition as compared to control. After 28 h, respiration rate declined in all the treatments up to 36 h albeit osmoprimed. At 32 h, the respiration rate was significantly higher in haloprimed seeds (30.3%) than other treatments but a significant difference was observed among all treatments at 36 h with control maintaining low rates. Further, a sharp increase of 29.7–76.0% was observed at 52 h in all the primed treatments as compared to control (Figure 4).

### Cytochrome c Oxidase Activity

**FIGURE 5:**
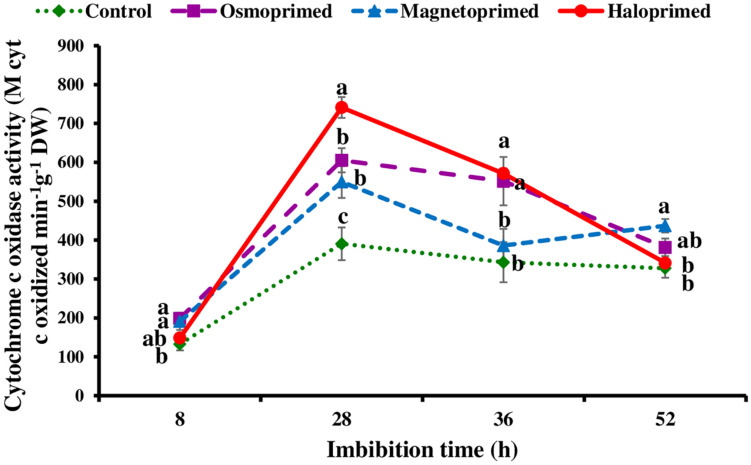
Cytochrome c oxidase activity of differently primed onion seeds at different imbibition time. Data represent mean ± SE of three biological replications. Different letters indicate significant differences among means based on LSD test (*p* < 0.05).

Cytochrome c oxidase activity was measured at 8, 28, 36, and 52 h of imbibition in response to the pattern of CO_2_ evolution rate i.e., increasing CO_2_ levels from 8–28 h followed by decreasing levels up to 36 h. At 28 h of imbibition, cytochrome c oxidase activity was significantly higher in haloprimed (89.8%) seed followed by osmo- (57.0%) and magneto- (40.7%) primed seeds compared to control. At 52 h of imbibition, haloprimed and unprimed seeds showed significantly low cytochrome c oxidase activities (Figure 5).

### HPLC Profile of Soluble Sugars

**FIGURE 6:**
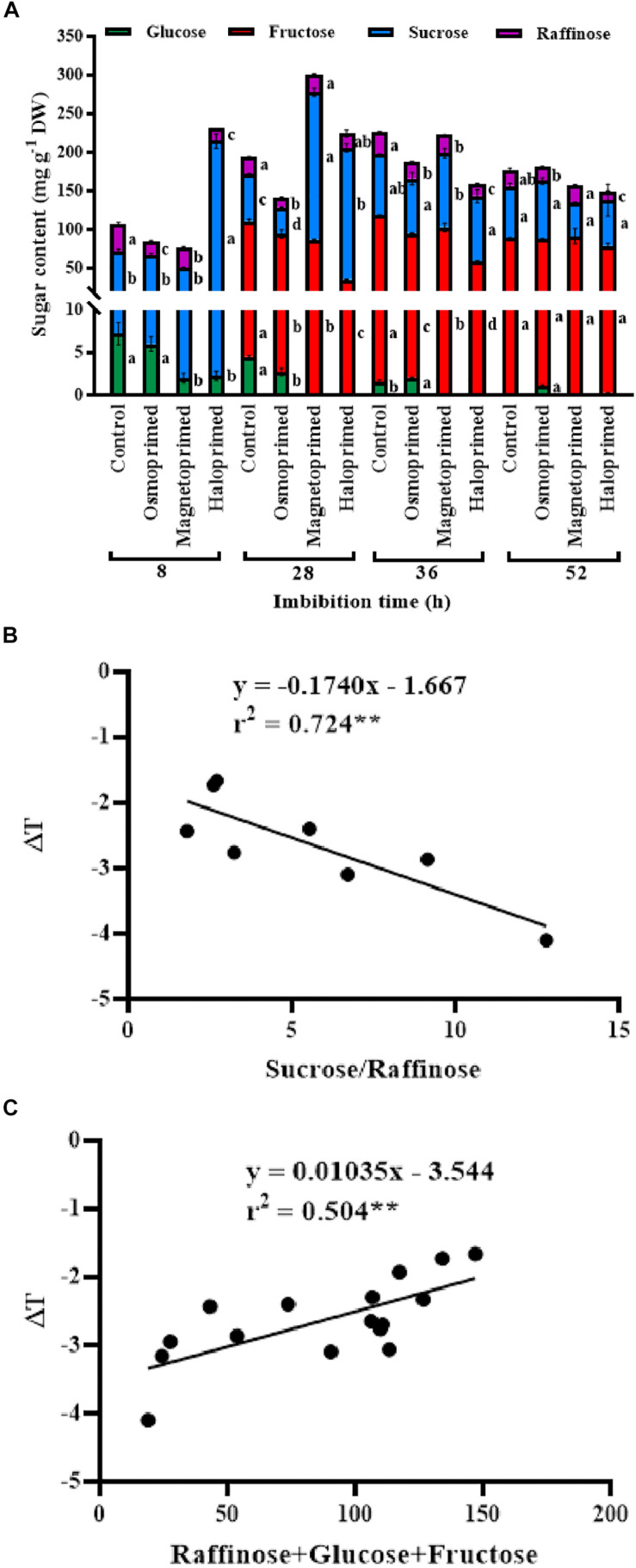
**(A)** Sugar profile of differently primed onion seeds at different time of imbibition. Data represent mean ± SE of three biological replications. Different letters indicate significant differences among means of each treatment at particular time of imbibition based on LSD test (*p* < 0.05). **(B)** Pearson’s correlation analysis between sucrose to raffinose ratio and relative temperatures (ΔT) of haloprimed and unprimed onion seeds. **(C)** Pearson’s correlation analysis between sum of raffinose, glucose, fructose, and relative temperatures (ΔT) of differently primed and unprimed onion seeds. Statistical significance: **p < 0.01 (two-tailed).

Sucrose, oligosaccharides of raffinose and fructose were the major sugars present in the germinating seed and constituted 24.0–75.0, 7.0–33.0, and 15–64% of the total sugars, respectively, at different treatments under different times of imbibition. Sucrose content was 3.3-fold higher in haloprimed seeds compared to control, at 8 h of imbibition which reduced at 28, 36, and 52 h of imbibition. Magnetoprimed seeds also showed increased sucrose levels at 28 h of imbibition (3.2-fold more than control) followed by a similar decline at 52 h. On the contrary, control and osmoprimed seeds did not show a similar trend in sucrose levels throughout the germination process. Raffinose content decreased in haloprimed seeds with the progress of germination. Of the two detected monosaccharides (glucose and fructose), fructose was 2.0–3.0-fold low in haloprimed seed compared to control at 28–36 h of imbibition but was undetectable in the initial (8 h) phase of germination. At 52 h, fructose content was significantly *at par* in all the treatments. Glucose levels were very less in all treatments. A decreasing trend in glucose content was observed in all the treatments with the advancement of germination. Sucrose to raffinose ratio followed the same trend as observed for the high respiration rates in the halo- and magneto-primed seeds at the onset of germination. The rate of utilization of sucrose was highest in haloprimed seeds at 8 h of imbibition (Figure 6).

## Discussion

In the present study, seed priming treatments did not show a significant change in percent germination but the emergence index, which is a measure of germination rate and vigor was 24.0–42.5% higher than unprimed ones. Our previous studies also showed the increased speed of germination in magnetoprimed tomato seeds under lab conditions (Anand et al., 2019). Seed priming increases the seed performance by metabolically progressing seed germination, which results in fast and uniform seedling emergence. Onion halopriming increased emergence index indicating an enhanced adaptation and positive response of primed seeds to soil stress conditions. The rate of germination also improved as seedlings emerged 5–6 days earlier than the control ones (De Souza et al., 2014). Similar findings have been reported in a review by Maffei (2014) wherein positive effects of priming were observed in maize (Florez et al., 2007), rice (Carbonell et al., 2011), barley (Martinez et al., 2000) and tomato (De Souza et al., 2010). The changes in the heat flow of seed during the germination process are affected by imbibition, respiration, decomposition of nutrients and other physical, biochemical and chemical reactions correlated with seed viability (Kim et al., 2013, 2014). The infrared thermography technique has a great potential in assessing the seed viability and vigor, as the measurement of a minor change in seed temperature (heat flow) can help in the evaluation of real-time metabolic changes in the germinating seed before seedling emergence (Kranner et al., 2010; Men et al., 2017). The relative temperature (ΔT) is the difference between the temperature of the seed and the surrounding medium and more pronounced ΔT values suggest that germinating seeds have much lower/higher temperature than the surrounding medium.

In general, negative values of ΔT were observed in all treatments, with primed seeds showing the higher temperature fluctuations during 8–32 h of water uptake. The thermogenic activities in the viable seed have been correlated with the kinetics of seed storage compounds in the germinating pea seeds by Kranner et al. (2010). They generated *in vitro* heat production and cooling properties of carbohydrates to simulate imbibed germinating seeds and reported positive heat of solution by high molecular-weight (HMW) carbohydrates like starch and the negative heat of solution by low molecular-weight (LMW) sugars like glucose, fructose, and sucrose and “raffinose” family. Since the germinating primed and unprimed seeds in our study showed negative heat of solution, we determined the profiles of LMW sugars in the germinating primed and control seeds. Haloprimed followed by magnetoprimed seeds that showed high emergence index also displayed more negative values of relative seed temperatures.

We found that glucose content decreased from 8–52 h of imbibition in all seeds. Halo- and magneto-primed seeds showed similar and very low glucose content at different times of imbibition. Monosaccharides like glucose which are present in mature seeds may serve as a primary source of energy when the seeds are kept in contact with water. Fructose could not be detected as early as 8 h of imbibition but was less in primed seeds at 28 h compared to control. The increase in sucrose content was more or less similar in the halo- and magneto-primed seeds which suggested the synthesis of sucrose before radicle emergence in these two efficient priming treatments. Sucrose is synthesized by the enzymatic action of sucrose phosphate synthase and is the translocable form of sugar required by the emerging seedling (Brown and Huber, 1987).

A positive correlation (*r*^2^ = 0.504^∗∗^) existed between the sum of raffinose, glucose and fructose content and relative temperatures of the seed. A higher amount of these three sugars led to less negative relative temperatures in control seeds compared to primed ones which may be due to delayed dissolution of these sugars. The cooling sugars are stored in orthodox seeds during seed maturation and form a cytoplasmic glass needed for their endurance in the dry state (Hoekstra, 2005). When the seed is kept for imbibition the sugars dissolve liberating ATP through the respiratory mechanism, thus lowering the temperature of the seed in the process (Kranner et al., 2010). The role of the breakdown of RFOs on germination in pea was tested by Blochl et al. (2007), by inhibiting the breakdown of RFOs on treating pea seeds with 1-deoxygalactonojirimycin (DGJ), an inhibitor of α-galactosidase activity and RFO catabolism. A significant delay in germination was observed that showed that RFO catabolism is required for early germination. In our study, higher content of raffinose along with glucose and fructose in control seeds pointed toward the less negative seed temperatures implying slower metabolic activity of control seeds. Obendorf (1997) identified that additional RFOs over a threshold limit of RFO may not increase the vigor. Li et al. (2017) observed the distinction between RFOs modulating seed vigor in monocot and dicot plants. They found a positive correlation between total RFOs, RFO/sucrose ratio, but not absolute individual RFO amounts (stachyose and verbascose contributing more than raffinose) and seed vigor in *Arabidopsis thaliana*, whereas in monocot maize RFO/sucrose ratio was responsible for high vigor. Our results contradicted their finding as we observed lower raffinose to sucrose ratio in haloprimed seeds throughout the germination period. On the other hand, a strong negative correlation (*r*^2^ = −0.724^∗∗^) was evident between sucrose/raffinose ratio and relative temperatures of the seed, indicating their role in the metabolism of primed seeds. Further studies on this observation can reveal the significance of this relationship in the primed seeds.

Seed respiration rate is one of the characteristic indicator of seed vigor that determines the performance of seed during germination and seedling emergence (Woodstock et al., 1984). Respiratory activity measured as the rate of carbon dioxide evolution due to breakdown of seed reserves was higher in primed seeds. High respiration rate and increased production of ATP stimulated the seedling growth of primed rice under chilling (Hussain et al., 2016). Our study showed that a low rate of respiration was linked to high levels of unutilized sugars, e.g., raffinose, glucose, and fructose in the respiratory process. This was dependent on the imbibition time at which the sugars were measured as significant correlation existed at 28 h (*r*^2^ = 0.419^∗^), which is the inflection point in the respiration curve. Moreland et al. (1974) reported that an increase in ATP content in germinating radish seeds corresponded with radicle emergence from the seeds. Siegenthaler and Douet-Orhant (1994) correlated ATP content with germination capacity of onion seeds only at specific 17 h of imbibition in comparison to their observations recorded at 4 and 7 h.

**FIGURE 7:**
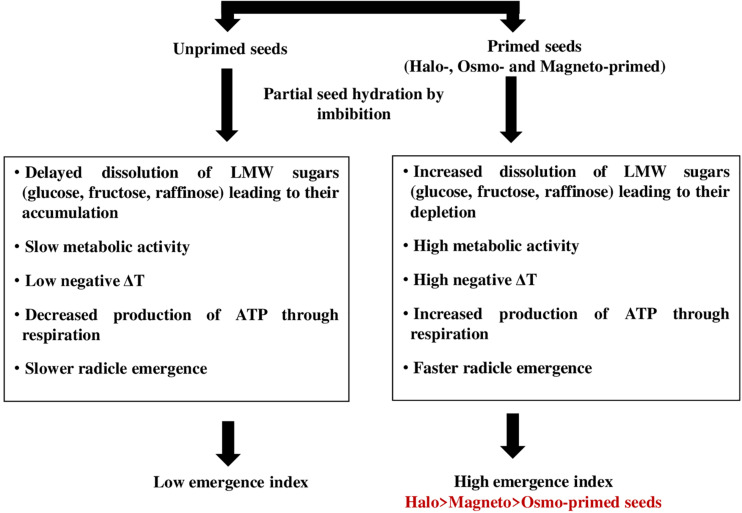
Schematic model explaining the high emergence index of primed seed is facilitated by faster dissolution of low molecular weight sugars (Raffinose + Glucose + Fructose) resulting in more negative IR thermal temperatures in actively metabolizing seeds.

The energy required for the initiation of the germination process may be provided through the cytochrome pathway. Cytochrome c oxidase is the last respiratory complex of the electron transfer chain in mitochondria and is responsible for transferring electrons to the final acceptor, i.e., oxygen in the classical respiratory pathway (Dahan et al., 2014). Liberated energy can be consumed for the transport of suprastoichiometrical protons across the mitochondrial membrane by cytochrome c oxidase. Increased activity of the cytochrome pathway was responsible for higher O_2_ uptake in soybean seed axes during the first 2–14 h of imbibition (Puntarulo et al., 1987). This energy is a by-product of carbohydrate breakdown where sucrose and RFOs formed the most important soluble sugars (Peterbauer and Richter, 2001). Increase in cytochrome c oxidase activity at 28 h in haloprimed seeds may be involved in the transport of electrons for ATP production, which are liberated from the breakdown of respiratory substrates like raffinose, glucose, and sucrose as they were detected in low amount and resulted in insignificantly more negative relative seed temperature. We propose a schematic model to explain that the high emergence index of primed seed is facilitated by the faster dissolution of low molecular weight sugars resulting in more negative IR thermal relative seed temperatures in actively metabolizing primed seed (Figure 7).

## Conclusion

Emergence index in onion seeds can be significantly improved by halopriming and magnetopriming treatments. The thermal fingerprints of the germinating primed seeds showed that of all priming treatments, haloprimed seeds possessed more negative relative seed temperatures which could be correlated with the cooling effect brought about by dissolution of low molecular weight sugars like raffinose, glucose and fructose.

## Data Availability Statement

The raw data supporting the conclusions of this article will be made available by the authors, without undue reservation.

## Author Contributions

AA conceptualized the hypothesis, designed the experiment, and interpreted the results. MT and AA prepared the manuscript. MT conducted the biochemical studies. PS analyzed the thermal fingerprints of imbibed seeds. AB and MT conducted the respiration experiment. SP conducted the HPLC studies. VP and AB helped in finalizing the manuscript. All authors contributed to the article and approved the submitted version.

## Conflict of Interest

The authors declare that the research was conducted in the absence of any commercial or financial relationships that could be construed as a potential conflict of interest.
